# Rural specialty care for Veterans with the chronic overlapping pain conditions: Fibromyalgia, migraine, or irritable bowel syndrome

**DOI:** 10.1111/jrh.70132

**Published:** 2026-03-16

**Authors:** Katherine Hadlandsmyth, Mary A. Driscoll, Jenna L. Adamowicz, Lauren Garvin, Brian C. Lund

**Affiliations:** ^1^ VA Office of Rural Health (ORH), Veterans Rural Health Resource Center‐Iowa City (VRHRC‐IC), Iowa City VA Health Care System Iowa City Iowa USA; ^2^ Center for Access & Delivery Research and Evaluation (CADRE), Iowa City VA Health Care System Iowa City Iowa USA; ^3^ College of Nursing University of Iowa Iowa City Iowa USA; ^4^ Pain Research, Informatics, Multimorbidities, and Education (PRIME) Center, VA Connecticut Healthcare System West Haven Connecticut USA; ^5^ Department of Psychiatry Yale School of Medicine New Haven Connecticut USA; ^6^ Department of Psychiatry University of Iowa Carver College of Medicine Iowa City Iowa USA; ^7^ Department of Epidemiology University of Iowa College of Public Health Iowa City Iowa USA

**Keywords:** chronic overlapping pain conditions, chronic pain, rural, specialty care, Veteran

## Abstract

**Purpose:**

This study examines primary and specialty health care among rural and urban Veterans with three common chronic overlapping pain conditions (COPCs): fibromyalgia, migraine, and irritable bowel syndrome (IBS) and the impact of both rural residence and rural primary care site on access to specialty care.

**Methods:**

The cohort included all Veterans treated for fibromyalgia, migraine, and/or IBS in the VA in 2022. The frequency of outpatient primary care and specialty care encounters for these COPCs in the following year was contrasted by residence (urban/rural) and primary care site (medical center, urban clinic, or rural clinic) using multivariate log‐binomial regression. Models were adjusted for demographics and comorbidities.

**Findings:**

250,533 Veterans were treated in the VA for the COPCs fibromyalgia, migraine, and/or IBS in 2022; 30.5% were rural residing. Relative to urban Veterans, rural Veterans were significantly more likely to have a primary care visit coded for a COPC (79.5% vs. 74.8%; *p* < 0.001) and less likely to have a specialty care visit coded for a COPC (31.8% vs. 37.8%; *p* < 0.001). After adjustment, rural residents were somewhat less likely to receive specialty care for their COPC, relative to urban residing Veterans (RR = 0.91, 95% CI: 0.90–0.92). Further, Veterans receiving care at urban clinics (RR = 0.81, 95% CI: 0.80–0.81) and rural clinics (RR = 0.66, 95% CI: 0.64–0.67) were substantially less likely to have a specialty care visit coded for a COPC, relative to larger VA medical centers. Rural/urban differences are also presented for a referent cohort of musculoskeletal pain conditions.

**Conclusions:**

Rural Veterans with COPCs may benefit from increased access to specialty pain care, which may also reduce burden on rural primary care providers.

## INTRODUCTION

Chronic pain is prevalent among US military Veterans.^1^, ^2^ Rural Veterans with chronic pain are at increased risk for potentially risky prescribing (i.e., long‐term opioids and central nervous system active medication polytherapy) and may face greater barriers to accessing comprehensive multidisciplinary pain care, relative to their urban counterparts.^3^, ^4^, ^5^, ^6^ The risks for suboptimal pain care may be magnified for the most complex rural patients with chronic pain.

Nociplastic pain is among the most severely disabling forms of chronic pain.^7^ Nociplastic pain has been defined by the International Association for the Study of Pain (2017), as “pain that arises from altered nociception despite no clear evidence of actual or threatened tissue damage causing the activation of peripheral nociceptors or evidence for disease or lesion of the somatosensory system causing pain.”^8^ In addition to chronic widespread pain, nociplastic pain typically presents as a constellation of central nervous system derived symptoms, including fatigue, gastrointestinal symptoms, genitourinary symptoms, headaches, concentration difficulties, sensory sensitivities, difficulties with sleep, and low mood.^7^ Nociplastic pain can be particularly challenging to treat and can result in diagnoses of chronic overlapping pain conditions (COPCs).^9^ The 10 COPCs include chronic migraine headache, fibromyalgia, irritable bowel syndrome (IBS), urologic chronic pelvic pain syndromes, vulvodynia, chronic tension‐type headache, temporomandibular disorder, myalgic encephalomyelitis/chronic fatigue syndrome, painful endometriosis, and chronic low back pain.^9^, ^10^, ^11^ Although these conditions commonly co‐occur, COPC is an overarching term referring to having at least one of the above conditions (i.e., not requiring two or more). For the current study, three index COPCs were chosen based on their prevalence, multi‐symptom presentation, and likelihood of being treated in specialty care. Specifically, fibromyalgia, migraine, and IBS were selected, as these are the most common COPCs among Veterans after lower back pain; however, unlike lower back pain, these three conditions are characterized by multi‐symptom presentations and thus more likely to represent the underlying unique mechanism of nociplastic pain.^7^, ^12^, ^13^, ^14^

Over 50 million people are affected by COPCs,^15^ which are associated with elevated depression, anxiety, high somatic symptom burden, and high rates of distress and pain catastrophizing.^9^, ^16^ Further, patients with COPCs tend to use high amounts of health care, including specialty care, and report low satisfaction with care.^9^ Rural Veterans with COPCs may face unique challenges in accessing needed pain care including poorly understood etiology, stigma, and limited availability of evidence‐based treatments and accessibility to specialists with knowledge of these conditions, or medical and mental health comorbidities.^7^, ^9^, ^10^, ^16^ Thus, rural Veterans with COPCs may face barriers in seeking appropriate care or understandably direct care to their local primary care provider, who may lack knowledge and/or ready access to evidence‐based multidisciplinary teams or interventions to manage complex patients with COPCs. However, it is currently unknown whether patterns of health care utilization among Veterans with COPCs differ between rural‐ and urban‐dwelling Veterans or between Veterans receiving care at different types of sites (i.e., large VA medical centers, urban and rural Community Based Outpatient Clinics: CBOCs).

The current study examines health care utilization in the VA among rural and urban Veterans with three of the most common COPCs that can each present with multiple symptoms and may benefit from specialty care: fibromyalgia, migraine, and IBS. The current study seeks to determine whether rates of primary and specialty care visits vary among these Veterans by the following: rural versus urban residence, site of care (i.e., medical centers, urban and rural CBOCs), and contrasted with Veterans with other non‐COPC musculoskeletal pain conditions. This will determine whether any rural/urban differences in utilization of primary and specialty care are unique to Veterans with COPCs, or if they also hold true in a referent cohort (i.e., those with non‐COPC musculoskeletal pain conditions).

## METHODS

### Data sources

National administrative data from the Veterans Health Administration were accessed from the VA Corporate Data Warehouse using the VA Informatics and Computing Infrastructure. Outpatient encounter data were used to identify Veterans with COPCs and musculoskeletal pain conditions, and to tabulate counts of primary and specialty care encounters coded for these diagnoses. These data were further used to assign primary care site and assess the presence of medical and psychiatric comorbidity. Geocoded enrollee files were used to determine patient residence (urban vs. rural). This study was reviewed by the local Institutional Review Board and determined not to constitute human subjects research and classified as exempt.

### Patients

The COPC cohort included Veterans receiving care for fibromyalgia, migraine, or IBS in the Veterans Health Administration during calendar year (CY) 2022. This required at least two outpatient encounters,^2^, ^17^ with the first occurring in CY22. To maximize the likelihood of capturing a treatment seeking population, and not exclude rural patients who may receive less frequent care, the second encounter coded for the same diagnosis had to occur at least 30 days, but less than 730 days, prior to the first encounter. International Classification of Diseases, 10th edition (ICD‐10) codes were as follows: fibromyalgia (M79.7), migraine (G43.x, excluding G43.6x and G43.Ax), and IBS (K58.0, K58.1, K58.2, K58.8, K58.9).^11^ For the IBS cohort, patients with evidence of inflammatory bowel disease were excluded; this included those with at least 2 outpatient encounters coded for Crohn's disease (K50.x) or ulcerative colitis (K51.x).^18^ Identical criteria were used to identify the musculoskeletal pain condition cohort, using ICD‐10 codes for neck, joint, or limb pain.^19^ Veterans with any of the 10 COPCs were excluded from the musculoskeletal cohort.

### Geographic variables

Geocoded resource files created by the VA Planning System Support Group Classification were used to classify Veteran residence as urban or rural. The VA classifies residences in areas with Rural‐Urban Commuting Areas (RUCA) codes 1.0 and 1.1 as urban and all other codes as rural.^20^ We defined a Veteran's primary care site based on the VA facility from which they received the greatest number of primary care encounters in CY22 and CY23. Primary care encounters were identified by VA clinic stop codes (170, 172, 176, 177, 178, 323, 342, 338, 348, 350) attached to outpatient encounters and, for the purpose of establishing care site, were not limited to encounters coded for a pain condition, but primary care encounters for any diagnosis. Identified primary sites site were then classified as either a large VA medical center or a clinic, where clinics where further classified as an urban clinic or a rural clinic using the zip code of the facility's location and the same RUCA categorization used for residence (urban: 1.0 and 1.1; rural: all others).

### Utilization metrics

Primary utilization metrics were the frequencies of any outpatient encounter, of specified types, coded for a target COPC diagnosis (fibromyalgia, migraine, or IBS) or non‐COPC musculoskeletal pain diagnosis (for the separate musculoskeletal cohort) during the year following (and including) the first observed COPC‐coded encounter during CY2022. Encounter types of interest were primary care encounters, identified by VA clinic stop codes (170, 172, 176, 177, 178, 323, 342, 338, 348, 350) and specialty care encounters for pain clinic (420), rheumatology (314), neurology (41, 293, 315, 325), gastrointestinal (33, 307), or mental health (500–599). These specialty areas were selected based on the types of specialty care typically used by patients with fibromyalgia, migraine, or IBS. As an alternative to frequency, counts of these encounters during the observation period were analyzed as secondary utilization metrics. Audio and video‐based telehealth encounters were included in addition to traditional face‐to‐face encounters.

### Analysis

Primary analyses were conducted with the combined cohort of Veterans with any one of the target COPCs (fibromyalgia, migraine, or IBS), with secondary analyses conducted for each diagnosis separately. COPC‐coded encounter frequencies for primary and specialty care were contrasted across residence and primary care site using chi‐square tests and encounter counts were contrasted using Wilcoxon rank sum tests. Multivariable log‐binomial regression was used to examine the independent associations of residence and primary care site with the likelihood of having received a specialty care encounter coded for a COPC, while further adjusting for Veteran demographics and mental health, medical, and COPC comorbidities. Mental health comorbidities included posttraumatic stress disorder, depressive disorder, substance use disorder, anxiety disorder, bipolar disorder, psychotic disorder, and sleep disorder.^21^ Medical comorbidity was assessed using the Charlson Comorbidity Index^22^ and other COPCs included temporomandibular disorders, vulvodynia, myalgic encephalomyelitis/chronic fatigue syndrome, urologic chronic pelvic pain syndrome, painful endometriosis, chronic tension‐type headache, and chronic lower back pain.^11^ A secondary multivariable analysis of encounter count as the dependent variable, rather than frequency, was conducted using negative binomial regression. Encounters coded for more than one COPC (e.g., fibromyalgia and migraine) were included in encounter counts for each individual diagnosis in secondary analyses but only counted as a single encounter in the primary analysis of the combined COPC cohort. Parallel analyses were conducted in the reference cohort of Veterans with musculoskeletal pain conditions. The primary intent of these analyses was to determine whether any relationships observed for utilization of primary and specialty care based on Veteran residence and primary care site were unique to Veterans with COPCs or also held true in this reference cohort. Bivariate comparisons in overall use of primary and specialty care between the musculoskeletal pain cohort and the COPC cohort were conducted with a chi‐square test. All statistical analyses were conducted using SAS version 9.4 and all tests were two‐tailed with α = 0.05.

## RESULTS

In 2022, a total of 250,533 Veterans were treated in the VA for the COPCs fibromyalgia, migraine, or IBS. The mean age of this COPC cohort was 49.0 years (Table 1). The cohort was 66.8% male, 64.9% White, 10.5% Hispanic, and 30.5% rural. Psychiatric and sleep disorder comorbidities were prevalent in this cohort, with the highest rates being 49% with diagnosed sleep disorders; 47.6%, depressive disorders; and 32.7%, posttraumatic stress disorder (Table 1). Patient characteristics for individual COPC diagnostic cohorts (i.e., fibromyalgia, migraine, and IBS separately) and for the comparator musculoskeletal pain cohort are presented in Table S1.

**TABLE 1:** Patient characteristics among Veterans in the chronic overlapping pain conditions (COPC) cohort, including fibromyalgia, migraine, or irritable bowel syndrome.

Characteristic	COPC cohort^*^
	*N* = 250,533
Age, mean (SD)	49.0 (14.1)
Age, *n* (%)	
< 45	105,701 (42.2)
45–54	60,739 (24.2)
55–64	45,331 (18.1)
≥ 65	38,762 (15.5)
Sex, *n* (%)	
Male	167,399 (66.8)
Female	83,134 (33.2)
Race, *n* (%)	
White	162,622 (64.9)
Black	60,013 (24.0)
Other	11,996 (4.8)
Unknown	15,902 (6.3)
Hispanic ethnicity, *n* (%)	26,383 (10.5)
Residence, *n* (%)	
Urban	174,079 (69.5)
Rural	76,454 (30.5)
Primary care site, *n* (%)	
Medical center	80,988 (32.3)
Urban clinic	143,172 (57.1)
Rural clinic	26,373 (10.5)
Comorbidities, *n* (%)	
Sleep disorder	122,671 (49.0)
Depression	119,373 (47.6)
Posttraumatic stress disorder	82,042 (32.7)
Substance use disorder	27,367 (10.9)
Generalized anxiety disorder	24,700 (9.9)
Bipolar disorder	11,542 (4.6)
Panic disorder	8068 (3.2)
Psychotic disorder	4043 (1.6)
Obsessive‐compulsive disorder	2138 (0.9)
Charlson comorbidity index, mean (SD)	0.8 (1.4)
COPCs, *n* (%)	
Fibromyalgia	30,462 (12.2)
Migraine	188,640 (75.3)
Irritable bowel syndrome	49,169 (19.6)
Other COPCs^**^	108,462 (43.3)

* Chronic overlapping pain condition (COPC) cohort includes fibromyalgia, migraine, and irritable bowel syndrome.

** Other COPCs include temporomandibular disorders, vulvodynia, myalgic encephalomyelitis/chronic fatigue syndrome, urologic chronic pelvic pain syndrome, painful endometriosis, chronic tension‐type headache, chronic lower back pain.

### Primary care visits for COPCs

Among the COPC cohort (i.e., all Veterans with fibromyalgia, migraine, or IBS), 76.2% received care for their COPC from primary care, including 70.8% of Veterans with fibromyalgia, 75.5% with Migraine, and 76.8% with IBS (Table 2). Veterans in the COPC cohort received a mean of 1.4 (SD = 1.3) primary care visits (Table S2). Rural Veterans in the COPC cohort were significantly more likely than urban Veterans to receive a primary care visit coded for a COPC (79.5% vs. 74.8%; *p* < 0.001).

**TABLE 2:** Frequency of any encounter coded for the pain diagnosis during the year following the CY22 index date, including primary care encounters, and specialty encounters for selected clinic types.

	Pain cohorts				
	Chronic overlapping pain conditions				
Encounter type	Fibromyalgia *N* = 30,462 *n* (%)	Migraine *N* = 188,640 *n* (%)	Irritable bowel Syndrome *N* = 49,169 *n* (%)	COPC cohort^*^ *N* = 250,533 *n* (%)	Musculoskeletal pain cohort^**^ *N* = 600,480 *n* (%)
Primary care	21,563 (70.8)	142,353 (75.5)	37,755 (76.8)	190,954 (76.2)	499,529 (83.2)
Specialty care	12,192 (40.0)	68,399 (36.3)	14,637 (29.8)	90,159 (36.0)	91,640 (15.3)
Pain	4079 (13.4)	6424 (3.4)	308 (0.6)	10,212 (4.1)	35,099 (5.8)
Gastroenterology	349 (1.1)	779 (0.4)	12,725 (25.9)	13,616 (5.4)	1293 (0.2)
Neurology	1414 (4.6)	55,903 (29.6)	232 (0.5)	56,657 (22.6)	17,151 (2.9)
Rheumatology	4549 (14.9)	496 (0.3)	242 (0.5)	5084 (2.0)	31,112 (5.2)
Mental health	4657 (15.3)	12,965 (6.9)	1922 (3.9)	18,690 (7.5)	14,746 (2.5)

* Chronic overlapping pain condition (COPC) cohort includes fibromyalgia, migraine, and irritable bowel syndrome.

**Musculoskeletal pain cohort includes musculoskeletal pain conditions of the neck, joint, or limb.

### Specialty care visits for COPCs

Over one‐third (36%) of Veterans in the COPC cohort received specialty care for their pain condition. Type of specialty care varied by COPC type, with the highest percentage of migraine patients being seen in neurology (29.6%) and the highest percentage of Veterans with IBS being seen in gastroenterology (25.9%: Table 2). Veterans with fibromyalgia, however, were seen in a range of specialty clinics (13.4% pain clinic, 14.9% rheumatology, 15.3% mental health clinic: Table 2).

### Rates of primary and specialty care visits for COPCs by rural versus urban residence

Rural Veterans in the COPC cohort had significantly higher rates of primary care visits and significantly lower rates of specialty care visits for their COPC, relative to urban Veterans (*p* <.001; Table 3). Only 31.8% of rural Veterans with COPCs, compared to 37.8% of urban Veterans with COPCs, had specialty care visits for their COPC (Table 3). Rural Veterans with COPCs had a mean of 0.90 (SD = 2.0) specialty care visits for their COPC, compared to 1.07 (SD = 2.2) for urban Veterans (*p* < 0.001).

**TABLE 3:** Encounters coded for a chronic overlapping pain condition (fibromyalgia, migraine, or irritable bowel syndrome), contrasted by urban and rural residence.

	Veteran residence		
Encounter type	Urban *N* = 174,079	Rural *N* = 76,454	Statistics
Any encounter	*n* (%)	*n* (%)	χ^2^; *p*‐value
Primary care	130,183 (74.8)	60,771 (79.5)	648; <0.001
Specialty care	65,881 (37.8)	24,278 (31.8)	855; <0.001
Count of encounters	Mean (SD)	Mean (SD)	*Z*; *p*‐value
Primary or specialty care	2.50 (2.5)	2.40 (2.3)	8.33; <0.001
Primary care	1.42 (1.3)	1.50 (1.3)	17.4; <0.001
Specialty care	1.07 (2.2)	0.90 (2.0)	28.5; <0.001

### Rates of COPC visits by site of care

When examined by clinic type (rural clinic, urban clinic, large VA medical center), Veterans with COPCs who received primary care from rural clinics were significantly more likely to be seen for their COPC in primary care (86.8%), relative to Veterans receiving primary care from an urban clinic (80.7%) or medical center (64.8%: Table 4). Further, Veterans who received primary care from rural clinics were also significantly less likely to be seen for their COPC by specialty care (25.5%), relative to Veterans with COPCs who received primary care from an urban clinic (33.8%) or large VA medical center (43.3%). Veterans with COPCs receiving primary care from rural clinics had a mean of 0.70 (SD = 1.8) specialty care visits for their COPC, compared to Veterans receiving primary care at urban clinics who had a mean of 0.95 (SD = 2.1) specialty care visits for their COPC, and Veterans receiving primary care from a large VA medical center who had a mean of 1.25 (SD = 2.3) specialty care visits for their COPC (*p* < 0.001).

**TABLE 4:** Encounter coded for a chronic overlapping pain condition (fibromyalgia, migraine, or irritable bowel syndrome), contrasted by primary care site (medical center, urban‐located clinic, or rural‐located clinic).

	Primary care site			Statistics	
Encounter type	Medical center *N* = 80,988	Urban clinic *N* = 143,172	Rural clinic *N* = 26,373	Urban clinic vs. medical center	Rural clinic vs. medical center
Any encounter	*n* (%)	*n* (%)	*n* (%)	χ^2^; *p*‐value	χ^2^; *p*‐value
Primary care	52,512 (64.8)	115,556 (80.7)	22,886 (86.8)	6950; <0.001	4580; <0.001
Specialty care	35,078 (43.3)	48,345 (33.8)	6736 (25.5)	2020; <0.001	2640; <0.001
Count of encounters	Mean (SD)	Mean (SD)	Mean (SD)	*Z*; *p*‐value	*Z*; *p*‐value
Primary or specialty	2.51 (2.7)	2.47 (2.4)	2.33 (2.1)	12.1; <0.001	1.17; 0.244
Primary care	1.25 (1.4)	1.52 (1.3)	1.63 (1.2)	65.6; <0.001	56.1; <0.001
Specialty care	1.25 (2.3)	0.95 (2.1)	0.70 (1.8)	44.6; <0.001	50.7; <0.001

### Multivariate analyses of COPC visit type by rural/urban residence and by site of care

Lower use of specialty care and increased reliance on primary care for the management of COPCs among rural residents, and those receiving care in rural clinics, was maintained in multivariate analyses, controlling for demographic variables, mental health comorbidities, medical comorbidities, and comorbid COPCs. Rural residing Veterans in the COPC cohort were significantly less likely to receive specialty care for their COPC, relative to urban residing Veterans (aRR = 0.91, 95% CI: 0.90 – 0.92: Table 5). When stratified by primary care site (medical center, urban clinic, or rural clinic), the lowest rates of specialty care for COPCs were seen among those receiving primary care from a rural clinic (aRR = 0.66, 95% CI: 0.64 – 0.67: Table 5). Similar patterns were found in sensitivity analyses using negative binomial regression models (Table S3).

**TABLE 5:** Likelihood of receiving specialty care coded for a chronic overlapping pain condition (fibromyalgia, migraine, or irritable bowel syndrome) using log binomial regression, contrasting residence (urban or rural) and primary care site (medical center, urban‐located clinic, or rural‐located clinic), *N* = 250,533.

Pain cohort	Unadjusted bivariate models	Adjusted multivariable model^*^
Residence and care site	RR (95% CI)	RR (95% CI)
Residence		
Urban	1.0 [Reference]	1.0 [Reference]
Rural	0.84 (0.83–0.85)	0.91 (0.90–0.92)
Primary care site		
Medical center	1.0 [Reference]	1.0 [Reference]
Urban clinic	0.78 (0.77–0.79)	0.81 (0.80–0.81)
Rural clinic	0.59 (0.58–0.60)	0.66 (0.64–0.67)

* Multivariable model was adjusted for demographics and mental health, medical, and COPC comorbidities.

### Examining individual COPCs

Trends in the use of primary and specialty care observed for the combined COPC cohort were also observed among individual COPCs (fibromyalgia, migraine, and IBS) when analyzed separately. A lower frequency of specialty care and increased reliance on primary care was observed among rural residents (Table S4) and patients of rural clinics (Table S5) among these pain cohorts, where findings remained significant after adjustment (Table S6). Parallel findings were further observed across individual COPC cohorts in sensitivity analyses examining encounters as a discrete count, rather than a frequency (Tables S7–S9).

### Comparing COPC care to non‐COPC musculoskeletal pain group

Finally, we included a cohort of patients with musculoskeletal pain conditions to serve as contrast against the COPC cohort in interpreting patterns of primary and specialty care service use. Overall rates of primary care were higher (83.2% vs. 76.2%; χ^2^ = 5610; *p* < 0.001) and overall rates of specialty care were lower (15.3% vs. 36.0%; χ^2^ = 45,200; *p* < 0.001) than the COPC cohort (Tables 2 and S2). However, the same patterns were seen of significantly higher rates of primary care and lower rates of specialty care among rural residents and among Veterans who received primary care from a rural clinic, both in terms of encounter frequency (Tables S4–S6) and encounter count (Tables S7–S9).

## DISCUSSION

Findings from the current study indicate that rural‐dwelling Veterans with fibromyalgia, migraine or IBS, and those receiving primary care from a rural CBOC, accessed significantly less specialty care relative to their urban counterparts, accompanied by an increased reliance on primary care. Differences based on Veteran residence were of modest clinical significance, where specialty care was accessed by 31.8% of rural residents, compared to 37.8% among urban residents, a difference of 9% after adjustment for confounding factors (OR = 0.91; 95% CI: 0.90–0.92). In contrast, differences based on care site were more pronounced and clinically meaningful, where only 25.5% of Veterans receiving primary care at rural clinics accessed specialty care, compared to 43.3%, a 34% difference after adjustment (OR = 0.66; 95% CI: 0.64–0.67). Of note, a similar pattern was found in the musculoskeletal cohort, suggesting a broader trend in rural pain care in the VA. Although a similar pattern was found among the musculoskeletal cohort, the higher overall rates of specialty care among the COPCs cohort may indicate differing pain care needs in this population. Our findings are consistent with prior qualitative work which has found that rural‐dwelling patients with COPCs (specifically fibromyalgia) may struggle to access needed health care resources, including providers with expertise in their pain conditions.^23^ Further, prior work has also demonstrated reduced access to pain specialty care among rural populations in general.^24^, ^25^, ^26^

The current findings also indicate that rural primary care providers are likely managing the majority of pain care for rural Veterans with COPCs. Given the potential clinical and psychosocial complexity in presenting concerns among this subset of rural Veterans with chronic pain (i.e., multiple pain conditions, high symptom severity, elevated rates of depression, anxiety, distress, and poor quality of life^9^, ^16^, ^27^), this may place undue burden on rural providers. Our results highlight the need for interventions at levels of both the health care system and the patient, with the combinatorial interplay of these viewed as necessary toward optimizing COPC care. Regarding the health care system, better resourcing for rural providers to support this patient population may reduce workplace stress, a known contributor to burnout among rural providers.^28^ Currently, there is a dearth of literature exploring both provider‐based impressions of their own education in COPC management, nor for what they would find helpful within such care visits (for example, e‐learning, toolkit, etc.). Providing accessible education on the nature of COPCs, rates, etiologies, and standards of care (with resources and accessible system referrals) for providers might support confidence and effectiveness within care visits. This may also impact implicit or explicit biases or stigma within the health care system that can impact patient care for those presenting with COPCs.

Rural Veterans with COPCs may also benefit from increased access to specialty pain care, which may again reduce burden on rural primary care providers. Specialty care clinics in the VA are typically located at major medical centers, which are predominantly in urban areas. To optimize Veterans access to evidence‐based treatment for COPCs, innovative pain care models may be needed. Specifically, rural Veterans may benefit from options for remote access to pain specialists and care coordinators. Models such as the TelePain program using a hub‐and‐spoke models^29^ or a telecollaborative care model, where a specialist nurse care manager coordinates pain care for rural Veterans,^30^ may prove critical to optimize care for rural Veterans with COPCs. Such programs may also increase specialty pain care utilization among rural Veterans.^24^

For rural Veterans themselves, patients may benefit from education on COPCs, tools for which have been successfully created and piloted in an electronic format that showed promise in increasing the educational component of self‐management (as opposed to the self‐management behaviors themselves).^31^ Combining patient self‐education materials with individualized treatment plans tailored to the specific patient might leverage increased interest or motivation in multidisciplinary pain treatment, including non‐pharmacological approaches and pain self‐management training. The combination of these health care systems (i.e., provider education, increased access to specialty providers via telecare options) and patient‐centered (equipping patients with empowering educational materials, providing them with access to specialists who can support them in biopsychosocial pain management behaviors) factors might serve to improve both patient and provider satisfaction with care, while also holding potential to impact the utilization metrics we explored here.^31^, ^32^

This study had some limitations. First, systems for classifying residence are imperfect and infrequently updated, which may produce inherent limitations in the dichotomous classification as urban versus rural. It is also possible that rural Veterans were receiving pain care through community resources (either paid for by the VA or private insurance), which were not included in the data for this study. We expect, however, that because higher rates of primary care were observed in conjunction with lower rates of specialty care among rural Veterans with COPCs, there is a different pattern of pain care emerging for rural Veterans, even with the absence of community pain care data. Further, these findings, which examined pain care within the VA, may not generalize to non‐VA health care settings. In addition, epidemiological methodologies based on medical record documentation of diagnoses do not confer diagnostic certainty. As such, caution may be warranted when using these data to interpret prevalence rates, however, these methods do facilitate wide‐scale examination of treatment seeking patterns in health services research. In addition, there is not an established threshold for minimal clinically important differences in number of visits, with which to compare the current findings. Finally, future work is needed to examine the reasons for the differences in primary and specialty pain care identified among rural Veterans with COPCs. There may be multiple factors contributing to this difference, such as access to specialty care, availability of telehealth options for primary and specialty care, clinician knowledge of recommended treatments, and/or patient interest in pursuing such treatments.

In conclusion, the current study suggests that rural primary care providers may be managing the majority of pain care for rural Veterans with COPCs. Rural Veterans with COPCs may benefit from innovative models of remotely delivered pain care, as well as increased access to specialty pain care. In addition to improving pain care for rural Veterans with COPCs, this may also reduce burden on rural primary care providers.

## CONFLICT OF INTEREST STATEMENT

There are no conflicts of interest to report for any authors.

## Supporting information



SUPPORTING INFORMATION
