# Supplementary material for: Rural specialty care for Veterans with the chronic overlapping pain conditions: Fibromyalgia, migraine, or irritable bowel syndrome
Source: J Rural Health. 2026 Mar 16;42(1):e70132. doi: 10.1111/jrh.70132 (PMC12992181; doi:10.1111/jrh.70132)
Supplement: Supplementary file 1 — SUPPORTING INFORMATION [file JRH-42-0-s001.docx]

Supplemental Table 1. Patient characteristics of individual chronic overlapping pain conditions (COPC)

|  | COPC cohorts | | |  |
| --- | --- | --- | --- | --- |
| Characteristic | Fibromyalgia  N=30,462 | Migraine  N=188,640 | Irritable bowel syndrome  N=49,169 | Musculoskeletal pain cohort*  N=600,480 |
| Age, mean (SD) | 53.7 (12.5) | 47.3 (13.3) | 52.4 (15.6) | 63.7 (14.8) |
| Age, n (%) |  |  |  |  |
| < 45 | 7,468 (24.5) | 88,041 (46.7) | 17,429 (35.4) | 75,246 (12.5) |
| 45-54 | 8,508 (27.9) | 46,670 (24.7) | 11,175 (22.7) | 71,484 (11.9) |
| 55-64 | 8,242 (27.1) | 31,904 (16.9) | 8,638 (17.6) | 126,764 (21.1) |
| ≥ 65 | 6,244 (20.5) | 22,025 (11.7) | 11,927 (24.3) | 326,986 (54.5) |
| Sex, n (%) |  |  |  |  |
| Male | 12,523 (41.1) | 126,313 (67.0) | 35,985 (73.2) | 549,723 (91.5) |
| Female | 17,939 (58.9) | 62,327 (33.0) | 13,184 (26.8) | 50,757 (8.5) |
| Race, n (%) |  |  |  |  |
| White | 20,818 (68.3) | 117,276 (62.2) | 36,251 (73.7) | 415,681 (69.2) |
| Black | 6,570 (21.6) | 49,363 (26.2) | 8,274 (16.8) | 128,622 (21.4) |
| Other | 1,318 (4.3) | 9,767 (5.2) | 1,713 (3.5) | 21,311 (3.5) |
| Unknown | 1,756 (5.8) | 12,234 (6.5) | 2,931 (6.0) | 34,866 (5.8) |
| Hispanic ethnicity, n (%) | 2,969 (9.7) | 21,250 (11.3) | 4,140 (8.4) | 43,309 (7.2) |
| Residence, n (%) |  |  |  |  |
| Urban | 20,400 (67.0) | 133,290 (70.7) | 32,782 (66.7) | 397,836 (66.3) |
| Rural | 10,062 (33.0) | 55,350 (29.3) | 16,387 (33.3) | 202,644 (33.7) |
| Primary care site, n (%) |  |  |  |  |
| Medical center | 10,843 (35.6) | 60,557 (32.1) | 15,793 (32.1) | 214,510 (35.7) |
| Urban clinic | 16,229 (53.3) | 109,182 (57.9) | 27,486 (55.9) | 315,204 (52.5) |
| Rural clinic | 3,390 (11.1) | 18,901 (10.0) | 5,890 (12.0) | 70,766 (11.8) |
| Comorbidities, n (%) |  |  |  |  |
| Sleep disorder | 16,010 (52.6) | 93,251 (49.4) | 23,262 (47.3) | 207,270 (34.5) |
| Depression | 17,827 (58.5) | 90,017 (47.7) | 22,196 (45.1) | 152,058 (25.3) |
| Posttraumatic stress disorder | 10,946 (35.9) | 63,447 (33.6) | 15,338 (31.2) | 85,031 (14.2) |
| Substance use disorder | 3,314 (10.9) | 19,873 (10.5) | 5,938 (12.1) | 61,181 (10.2) |
| Generalized anxiety disorder | 3,668 (12.0) | 17,918 (9.5) | 5,590 (11.4) | 23,873 (4.0) |
| Bipolar disorder | 2,080 (6.8) | 8,283 (4.4) | 2,435 (5.0) | 12,953 (2.2) |
| Panic disorder | 1,255 (4.1) | 5,931 (3.1) | 1,808 (3.7) | 6,790 (1.1) |
| Psychotic disorder | 558 (1.8) | 2,906 (1.5) | 865 (1.8) | 9,098 (1.5) |
| Obsessive-compulsive disorder | 354 (1.2) | 1,506 (0.8) | 541 (1.1) | 2,022 (0.3) |
| Other COPC** | 14,709 (48.3) | 82,984 (44.0) | 19,598 (39.9) | 124,146 (20.7) |
| Charlson comorbidity index, mean (SD) | 1.1 (1.6) | 0.7 (1.3) | 0.9 (1.6) | 1.5 (2.1) |

* Musculoskeletal pain cohort includes musculoskeletal pain conditions of the neck, joint, or limb.

** Other COPCs include: temporomandibular disorders, vulvodynia, myalgic encephalomyelitis/chronic fatigue syndrome, urologic chronic pelvic pain syndrome, endometriosis, chronic tension-type headache, chronic lower back pain.

Supplemental Table 2. Mean encounter counts coded for the pain diagnosis during the year following the CY22 index date, including total encounters, primary care encounters, and specialty encounters for selected clinic types.

|  | Pain cohorts | | | | |
| --- | --- | --- | --- | --- | --- |
|  | Chronic overlapping pain conditions | | | |  |
| Encounter type | Fibromyalgia  N=30,462  Mean (SD) | Migraine  N=188,640  Mean (SD) | Irritable bowel syndrome  N=49,169  Mean (SD) | COPC cohort*  N=250,533  Mean (SD) | Musculoskeletal pain cohort**  N=600,480  Mean (SD) |
| Primary and specialty | 2.7 (3.0) | 2.3 (2.1) | 1.9 (1.6) | 2.5 (2.4) | 2.3 (2.0) |
| Primary care | 1.4 (1.4) | 1.3 (1.2) | 1.3 (1.1) | 1.4 (1.3) | 1.9 (1.6) |
| Specialty care | 1.3 (2.7) | 1.0 (1.9) | 0.6 (1.3) | 1.0 (2.1) | 0.4 (1.4) |
| Pain | 0.4 (1.7) | 0.1 (0.7) | 0.0 (0.2) | 0.1 (0.9) | 0.2 (0.9) |
| Gastroenterology | 0.0 (0.1) | 0.0 (0.1) | 0.5 (0.9) | 0.1 (0.5) | 0.0 (0.1) |
| Neurology | 0.1 (0.4) | 0.7 (1.4) | 0.0 (0.1) | 0.6 (1.3) | 0.0 (0.3) |
| Rheumatology | 0.3 (0.9) | 0.0 (0.1) | 0.0 (0.1) | 0.0 (0.3) | 0.1 (0.6) |
| Mental health | 0.5 (1.8) | 0.2 (1.0) | 0.1 (0.8) | 0.2 (1.2) | 0.1 (0.8) |

* Chronic overlapping pain condition (COPC) cohort includes fibromyalgia, migraine, and irritable bowel syndrome.

**Musculoskeletal pain cohort includes musculoskeletal pain conditions of the neck, joint, or limb.

Supplemental Table 3. Likelihood of receiving specialty care coded for a chronic overlapping pain condition (fibromyalgia, migraine, or irritable bowel syndrome) using negative binomial, contrasting residence (urban or rural) and primary care site (medical center, urban-located clinic, or rural-located clinic), N=250,533.

| Pain cohort  Residence and care site | Unadjusted bivariate models  IRR (95% CI) | Adjusted multivariable model*  aIRR (95% CI) |
| --- | --- | --- |
| Residence |  |  |
| Urban | 1.0 [Reference] | 1.0 [Reference] |
| Rural | 0.84 (0.82-0.85) | 0.92 (0.90-0.94) |
| Primary care site |  |  |
| Medical center | 1.0 [Reference] | 1.0 [Reference] |
| Urban clinic | 0.76 (0.74-0.77) | 0.77 (0.76-0.78) |
| Rural clinic | 0.56 (0.54-0.57) | 0.60 (0.58-0.62) |

* Multivariable model was adjusted for demographics and mental health, medical, and COPC comorbidities.

Supplemental Table 4. Frequency of any encounter coded for the pain cohort diagnosis, contrasted by urban and rural residence.

|  | Veteran residence | |  |
| --- | --- | --- | --- |
| Pain cohort  Encounter type | Urban  n (%) | Rural  n (%) | Statistics  χ^2^; p-value |
| Individual COPCs |  |  |  |
| Fibromyalgia | N=20,400 | N=10,062 |  |
| Primary care | 13,955 (68.4) | 7,608 (75.6) | 169; <0.001 |
| Specialty care | 8,583 (42.1) | 3,609 (35.9) | 108; <0.001 |
| Migraine | N=133,290 | N=55,350 |  |
| Primary care | 98,928 (74.2) | 43,425 (78.5) | 379; <0.001 |
| Specialty care | 50,311 (37.7) | 18,088 (32.7) | 434; <0.001 |
| Irritable bowel syndrome | N=32,782 | N=16,387 |  |
| Primary care | 24,512 (74.8) | 13,243 (80.8) | 224; <0.001 |
| Specialty care | 10,740 (32.8) | 3,897 (23.8) | 421; <0.001 |
| Musculoskeletal pain cohort* | N=397,836 | N=202,644 |  |
| Primary care | 325,305 (81.8) | 174,224 (86.0) | 1700; <0.001 |
| Specialty care | 64,552 (16.2) | 27,088 (13.4) | 848; <0.001 |

COPC = Chronic Overlapping Pain Condition

*Musculoskeletal pain cohort includes musculoskeletal pain conditions of the neck, joint, or limb.

Supplemental Table 5. Frequency of any encounter coded for the pain diagnosis, contrasted by primary care site (medical center, urban-located clinic, or rural-located clinic).

|  | Primary care site | | | Statistics |  |
| --- | --- | --- | --- | --- | --- |
| Pain cohort  Encounter type | Medical center  n (%) | Urban clinic  n (%) | Rural clinic  n (%) | Urban vs MC  χ^2^; p-value | Rural vs MC  χ^2^; p-value |
| Individual COPCs |  |  |  |  |  |
| Fibromyalgia | N=10,843 | N=16,229 | N=3,390 |  |  |
| Primary care | 6,338 (58.5) | 12,340 (76.0) | 2,885 (85.1) | 940; <0.001 | 804; <0.001 |
| Specialty care | 4,823 (44.5) | 6,293 (38.8) | 1,076 (31.7) | 87.4; <0.001 | 173; <0.001 |
| Migraine | N=60,557 | N=109,182 | N=18,901 |  |  |
| Primary care | 38,603 (63.7) | 87,536 (80.2) | 16,214 (85.8) | 5510; <0.001 | 3270; <0.001 |
| Specialty care | 26,623 (44.0) | 36,828 (33.7) | 4,918 (26.2) | 1740; <0.001 | 1900; <0.001 |
| IBS | N=15,793 | N=27,486 | N=5,890 |  |  |
| Primary care | 10,548 (66.8) | 22,012 (80.1) | 5,195 (88.2) | 952; <0.001 | 989; <0.001 |
| Specialty care | 5,752 (36.4) | 7,845 (28.5) | 1,040 (17.7) | 289; <0.001 | 702; <0.001 |
| Musculoskeletal pain cohort* | | | | | |
|  | N=214,510 | N=315,204 | N=70,766 |  |  |
| Primary care | 166,704 (77.7) | 269,326 (85.4) | 63,499 (89.7) | 5240; <0.001 | 4930; <0.001 |
| Specialty care | 37,669 (17.6) | 45,974 (14.6) | 7,997 (11.3) | 850; <0.001 | 1550; <0.001 |

COPC=chronic overlapping pain condition; IBS=irritable bowel syndrome; MC=medical center

* Musculoskeletal pain cohort includes musculoskeletal pain conditions of the neck, joint, or limb.

Supplemental Table 6. Likelihood of receiving specialty care for target pain cohort diagnosis using log-binomial regression, contrasting residence (urban or rural) and primary care site (medical center, urban-located clinic, or rural-located clinic).

| Pain cohort  Residence and care site | Unadjusted bivariate models  RR (95% CI) | Adjusted multivariable model*  aRR (95% CI) |
| --- | --- | --- |
| Fibromyalgia; N=30,462 |  |  |
| Residence |  |  |
| Urban | 1.0 [Reference] | 1.0 [Reference] |
| Rural | 0.85 (0.83-0.88) | 0.93 (0.90-0.96) |
| Primary care site |  |  |
| Medical center | 1.0 [Reference] | 1.0 [Reference] |
| Urban clinic | 0.87 (0.85-0.90) | 0.90 (0.87-0.92) |
| Rural clinic | 0.71 (0.68-0.75) | 0.80 (0.76-0.85) |
| Migraine; N=188,640 |  |  |
| Residence |  |  |
| Urban | 1.0 [Reference] | 1.0 [Reference] |
| Rural | 0.87 (0.85-0.88) | 0.92 (0.91-0.94) |
| Primary care site |  |  |
| Medical center | 1.0 [Reference] | 1.0 [Reference] |
| Urban clinic | 0.77 (0.76-0.78) | 0.80 (0.79-0.81) |
| Rural clinic | 0.60 (0.58-0.61) | 0.65 (0.63-0.67) |
| Irritable bowel syndrome; N=49,169 |  |  |
| Residence |  |  |
| Urban | 1.0 [Reference] | 1.0 [Reference] |
| Rural | 0.73 (0.70-0.75) | 0.85 (0.82-0.87) |
| Primary care site |  |  |
| Medical center | 1.0 [Reference] | 1.0 [Reference] |
| Urban clinic | 0.78 (0.76-0.81) | 0.80 (0.78-0.82) |
| Rural clinic | 0.48 (0.46-0.51) | 0.58 (0.54-0.61) |
| Musculoskeletal pain**; N=600,480 |  |  |
| Residence |  |  |
| Urban | 1.0 [Reference] | 1.0 [Reference] |
| Rural | 0.82 (0.81-0.83) | 0.88 (0.86-0.89) |
| Primary care site |  |  |
| Medical center | 1.0 [Reference] | 1.0 [Reference] |
| Urban clinic | 0.83 (0.82-0.84) | 0.86 (0.85-0.87) |
| Rural clinic | 0.64 (0.63-0.66) | 0.72 (0.70-0.74) |

* Multivariable models were adjusted for demographics and mental health, medical, and COPC comorbidities.

** Musculoskeletal pain cohort includes musculoskeletal pain conditions of the neck, joint, or limb.

Supplemental Table 7. Mean encounter counts coded for the pain cohort diagnosis, contrasted by urban and rural residence.

|  | Veteran residence | |  |
| --- | --- | --- | --- |
| Pain cohort  Encounter type | Urban  Mean (SD) | Rural  Mean (SD) | Statistics  Z; p-value |
| Individual COPCs |  |  |  |
| Fibromyalgia | N=20,400 | N=10,062 |  |
| Primary or specialty care | 2.69 (3.1) | 2.57 (2.8) | 0.44; 0.660 |
| Primary care | 1.34 (1.4) | 1.47 (1.4) | 9.71; <0.001 |
| Specialty care | 1.35 (2.8) | 1.11 (2.6) | 10.3; <0.001 |
| Migraine | N=133,290 | N=55,350 |  |
| Primary or specialty care | 2.37 (2.1) | 2.29 (2.0) | 7.21; <0.001 |
| Primary care | 1.33 (1.2) | 1.39 (1.1) | 12.3; <0.001 |
| Specialty care | 1.04 (1.9) | 0.90 (1.9) | 20.3; <0.001 |
| Irritable bowel syndrome | N=32,782 | N=16,387 |  |
| Primary or specialty care | 1.95 (1.6) | 1.87 (1.4) | 3.79; <0.001 |
| Primary care | 1.31 (1.1) | 1.40 (1.1) | 11.1; <0.001 |
| Specialty care | 0.64 (1.4) | 0.47 (1.1) | 20.3; <0.001 |
| Musculoskeletal pain cohort* | N=397,836 | N=202,644 |  |
| Primary or specialty care | 2.25 (2.1) | 2.32 (1.9) | 30.8; <0.001 |
| Primary care | 1.83 (1.6) | 1.99 (1.6) | 49.4; <0.001 |
| Specialty care | 0.42 (1.5) | 0.33 (1.2) | 29.3; <0.001 |

COPC = Chronic Overlapping Pain Condition

* Musculoskeletal pain cohort includes musculoskeletal pain conditions of the neck, joint, or limb.

Supplemental Table 8. Mean encounter counts coded for the pain diagnosis, contrasted by primary care site (medical center, urban-located clinic, or rural-located clinic).

|  | Primary care site | | | Statistics |  |
| --- | --- | --- | --- | --- | --- |
| Pain cohort  Encounter type | Medical center  Mean (SD) | Urban clinic  Mean (SD) | Rural clinic  Mean (SD) | Urban vs MC  Z; p | Rural vs MC  Z; p |
| Individual COPCs |  |  |  |  |  |
| Fibromyalgia | N=10,843 | N=16,229 | N=3,390 |  |  |
| Primary or specialty | 2.64 (3.3) | 2.67 (2.9) | 2.59 (2.6) | 9.61; <0.001 | 6.57; <0.001 |
| Primary care | 1.18 (1.6) | 1.46 (1.3) | 1.64 (1.4) | 25.6; <0.001 | 24.3; <0.001 |
| Specialty care | 1.46 (2.9) | 1.21 (2.7) | 0.95 (2.3) | 9.62; <0.001 | 13.2; <0.001 |
| Migraine | N=60,557 | N=109,182 | N=18,901 |  |  |
| Primary or specialty | 2.37 (2.3) | 2.35 (2.0) | 2.21 (1.8) | 9.66; <0.001 | 0.69; 0.490 |
| Primary care | 1.15 (1.2) | 1.43 (1.1) | 1.51 (1.0) | 60.3; <0.001 | 48.3; <0.001 |
| Specialty care | 1.22 (2.1) | 0.93 (1.9) | 0.70 (1.6) | 41.0; <0.001 | 42.9; <0.001 |
| IBS | N=15,793 | N=27,486 | N=5,890 |  |  |
| Primary or specialty | 1.94 (1.8) | 1.93 (1.5) | 1.85 (1.3) | 5.77; <0.001 | 1.72; 0.085 |
| Primary care | 1.21 (1.3) | 1.38 (1.0) | 1.51 (1.0) | 22.9; <0.001 | 24.8; <0.001 |
| Specialty care | 0.73 (1.4) | 0.55 (1.3) | 0.34 (1.0) | 17.3; <0.001 | 26.3; <0.001 |
| Musculoskeletal pain cohort* | | | | | |
|  | N=214,510 | N=315,204 | N=70,766 |  |  |
| Primary or specialty | 2.28 (2.4) | 2.25 (1.9) | 2.37 (1.8) | 29.9; <0.001 | 42.9; <0.001 |
| Primary care | 1.81 (1.8) | 1.88 (1.4) | 2.10 (1.5) | 50.4; <0.001 | 69.1; <0.001 |
| Specialty care | 0.47 (1.6) | 0.37 (1.3) | 0.28 (1.1) | 29.5; <0.001 | 39.5; <0.001 |

COPC = chronic overlapping pain condition; IBS=irritable bowel syndrome; MC = medical center

* Musculoskeletal pain cohort includes musculoskeletal pain conditions of the neck, joint, or limb.

Supplemental Table 9. Likelihood of receiving specialty care for the target pain diagnosis using negative binomial regression, contrasting residence (urban or rural) and primary care site (medical center, urban-located clinic, or rural-located clinic).

| Pain cohort  Residence and care site | Unadjusted bivariate models  IRR (95% CI) | Adjusted multivariable model*  aIRR (95% CI) |
| --- | --- | --- |
| Fibromyalgia; N=30,462 |  |  |
| Residence |  |  |
| Urban | 1.0 [Reference] | 1.0 [Reference] |
| Rural | 0.82 (0.78-0.86) | 0.88 (0.84-0.93) |
| Primary care site |  |  |
| Medical center | 1.0 [Reference] | 1.0 [Reference] |
| Urban clinic | 0.83 (0.79-0.87) | 0.83 (0.80-0.87) |
| Rural clinic | 0.65 (0.60-0.71) | 0.73 (0.67-0.79) |
| Migraine; N=188,640 |  |  |
| Residence |  |  |
| Urban | 1.0 [Reference] | 1.0 [Reference] |
| Rural | 0.87 (0.85-0.89) | 0.94 (0.92-0.96) |
| Primary care site |  |  |
| Medical center | 1.0 [Reference] | 1.0 [Reference] |
| Urban clinic | 0.76 (0.74-0.77) | 0.77 (0.75-0.78) |
| Rural clinic | 0.57 (0.55-0.59) | 0.59 (0.57-0.61) |
| Irritable bowel syndrome; N=49,169 |  |  |
| Residence |  |  |
| Urban | 1.0 [Reference] | 1.0 [Reference] |
| Rural | 0.73 (0.70-0.76) | 0.85 (0.82-0.89) |
| Primary care site |  |  |
| Medical center | 1.0 [Reference] | 1.0 [Reference] |
| Urban clinic | 0.75 (0.72-0.78) | 0.76 (0.73-0.79) |
| Rural clinic | 0.47 (0.44-0.50) | 0.54 (0.51-0.58) |
| Musculoskeletal pain cohort***; N=600,480 | | |
| Residence |  |  |
| Urban | 1.0 [Reference] | 1.0 [Reference] |
| Rural | 0.79 (0.78-0.81) | 0.83 (0.82-0.85) |
| Primary care site |  |  |
| Medical center | 1.0 [Reference] | 1.0 [Reference] |
| Urban clinic | 0.79 (0.78-0.81) | 0.81 (0.80-0.83) |
| Rural clinic | 0.60 (0.58-0.62) | 0.68 (0.65-0.70) |

* Multivariable models were adjusted for demographics and mental health, medical, and Chronic Overlapping Pain Condition (COPC) comorbidities.

** COPC cohort includes fibromyalgia, migraine, or irritable bowel syndrome.

** Musculoskeletal pain cohort includes musculoskeletal pain conditions of the neck, joint, or limb.
